# KalmanFormer: using transformer to model the Kalman Gain in Kalman Filters

**DOI:** 10.3389/fnbot.2024.1460255

**Published:** 2025-01-07

**Authors:** Siyuan Shen, Jichen Chen, Guanfeng Yu, Zhengjun Zhai, Pujie Han

**Affiliations:** ^1^School of Computer Science, Northwestern Polytechnical University, Xi'an, China; ^2^Fourth Technical Department, Xi'an Microelectronics Technology Institute, Xi'an, China; ^3^Research Office 16, AVIC Xi'an Aeronautics Computing Technique Research Institute, Xi'an, China; ^4^Software Engineering College, Zhengzhou University of Light Industry, Zhengzhou, China

**Keywords:** Kalman Filter, deep learning, transformer, Kalman Gain, supervised paradigm

## Abstract

**Introduction:**

Tracking the hidden states of dynamic systems is a fundamental task in signal processing. Recursive Kalman Filters (KF) are widely regarded as an efficient solution for linear and Gaussian systems, offering low computational complexity. However, real-world applications often involve non-linear dynamics, making it challenging for traditional Kalman Filters to achieve accurate state estimation. Additionally, the accurate modeling of system dynamics and noise in practical scenarios is often difficult. To address these limitations, we propose the KalmanFormer, a hybrid model-driven and data-driven state estimator. By leveraging data, the KalmanFormer promotes the performance of state estimation under non-linear conditions and partial information scenarios.

**Methods:**

The proposed KalmanFormer integrates classical Kalman Filter with a Transformer framework. Specifically, it utilizes the Transformer to learn the Kalman Gain directly from data without requiring prior knowledge of noise parameters. The learned Kalman Gain is then incorporated into the standard Kalman Filter workflow, enabling the system to better handle non-linearities and model mismatches. The hybrid approach combines the strengths of data-driven learning and model-driven methodologies to achieve robust state estimation.

**Results and discussion:**

To evaluate the effectiveness of KalmanFormer, we conducted numerical experiments in both synthetic and real-world dataset. The results demonstrate that KalmanFormer outperforms the classical Extended Kalman Filter (EKF) in the same settings. It achieves superior accuracy in tracking hidden states, demonstrating resilience to non-linearities and imprecise system models.

## 1 Introduction

It is the most fundamental task to track the hidden state of a dynamical system by using the noisy measurements in real-time in many fileds, including singal processing (Yadav et al., 2023), navigation (Hu et al., 2003), information fusion (Xu et al., 2004), and automation control (Menner et al., 2023; Mercorelli, 2012a). A large number of algorithms were proposed to stress this issue, such as Bayesian estimation (Coué et al., 2003) and particle filter (Hue et al., 2002).

Kalman Filter (KF) (Kalman, 1960) is also an efficient recursive filter that can track the state of dynamic systems from a series of incomplete measurements with additive white Gaussian noise (AWGN). Low complexity implementation of KF, combined with theoretical foundation, resulted in it quickly becoming the popular method for state estimation problems.

The original Kalman Filters perform well in linear and Gaussian systems. The reality is that many nonlinear phenomena are encountered in real-world multi-sensor systems. Therefore, several variants of Kalman Filters are available to meet the requirements of nonlinear dynamic systems, including Extended Kalman Filters (Maybeck, 1982) (EKF) and Unscented Kalman Filters (UKF) (Wan and Van Der Merwe, 2001).

There are still limitations associated with the application of EKF and UKF in practical applications. Specifically, the Kalman Filter is a model-based method, and the performance of state estimation heavily depends on model accuracy. Furthermore, the noise covariance matrix is determined by prior process noise and measurement noise, which are assumed to be Additive White Gaussian Noise (AWGN). Additionally, there is no guarantee that the AWGN will accurately reflect the actual performance of the information fusion.

Several variants of Kalman Filters were proposed to overcome the above issue. For example, Huang et al. (2020) introduced a sliding window variational adaptive Kalman filter to simultaneously modify the state estimation and covariance matrix. Yu and Li (2021) presented an adaptive Kalman Filter that concentrated on unknown covariances of both dynamic multiplicative noise and additive noises. Xiong et al. (2020) employed a parallel adaptive Kalman Filter to estimate the attitude of the vehicle based on the Inertial Measurement Unit (IMU). Paolo Mercorelli introduced (Mercorelli, 2012b) a combination of the augmented EKF and EKF for sensorless Valve Control which avoids complicated observation.

Recent years have seen the application of deep learning techniques to multiple real-world applications such as computer vision (Voulodimos et al., 2018) and natural language processing (Otter et al., 2020). Particularly, some Deep Neural Networks (DNNs), such as the Recurrent Neural Network (RNN) (Elman, 1990), Long Short-Term Memory Network (LSTM) (Hochreiter and Schmidhuber, 1997), Gated Recurrent Unit (GRU) (Chung et al., 2014), and Transformer (Vaswani et al., 2017), have demonstrated excellent performance when it comes to processing time series data. For example, Xia et al. (2021) presented staked GRU and RNN to predict the payload of electricity. Zhang et al. (2020) applied LSTM to estimate the battery's state of health.

Furthermore, deep learning techniques have been utilized by some researchers to enhance the effects of the Kalman Filters. For example, Rangapuram et al. (2018) introduced RNN to forecast the state space parameters of linear systems. Coskun et al. (2017) utilized LSTM to learn the noisy parameters and motion model of the Kalman filters. EKFNet (Xu and Niu, 2021) used BPTT (Ruder, 2016) to learn the process and measurement noise from the measurement. Bence Zsombor Hadlaczky applied neural networks and EKF to estimate the wing shape (Hadlaczky et al., 2023). Dahal et al. (2024) introduced RobustStateNet, which applied RNN and Kalman Filters to perform ego vehicle state estimation. Zhang et al. (2023) adopted the Transformer to pre-estimate the vehicle mass, thus acting as an observation for EKF. Luttmann and Mercorelli (2021) employed EKF to accelerate the convergence of the learning system.

In this work, we present KalmanFormer, a hybrid data-driven and model state estimator that can be used to perform information fusion in multi-sensor systems. Our KalmanFormer uses a Transformer framework to track the Kalman Gain instead of computing it from the statistic moments.

The structure of this paper is organized as follows: Section 2 introduces the Kalman Filters and Transformer architecture. Section 3 details the methodology of the proposed KalmanFormer. Experiments will be discussed in Section 4. Section 5 concludes the whole paper.

## 2 Preliminary knowledge

### 2.1 Kalman Filter

The Kalman Filter algorithm (KF) is a classic algorithm of information fusion technology and is widely used to solve various optimal estimation problems. The classic Kalman Filters are composed of a state transition model and an observation model, which are expressed as follows:


(1) $$\{\begin{array}{l} x_{k}={\text{F}}_{k}x_{k-1}+{\text{B}}_{\text{k}}u_{k-1}+w_{k-1}\\ z_{k}={\text{H}}_{\text{k}}x_{k}+v_{k}\\ w_{k-1}\sim N(0,{\text{Q}}_{k})\\ v_{k}\sim N(0,{\text{R}}_{k}) \end{array}$$

where *x*_*k*_ is the state vector of the system, *F*_*k*_ represents the state transition matrix, *B*_*k*_ is the control-input model which is applied to the control vector *u*_*k*−1_, and *H*_*k*_ represents the observation function, which maps the true state space into the observed space. *w*_*k*−1_ and *v*_*k*_ are process noise and observation noises respectively. Process noise is assumed to be drawn from a zero multivariate normal distribution N with covariance **Q**_*k*_. Observation noise is assumed to be zero mean Gaussian white noise with covariance *R*_*k*_.

**Figure 1:**
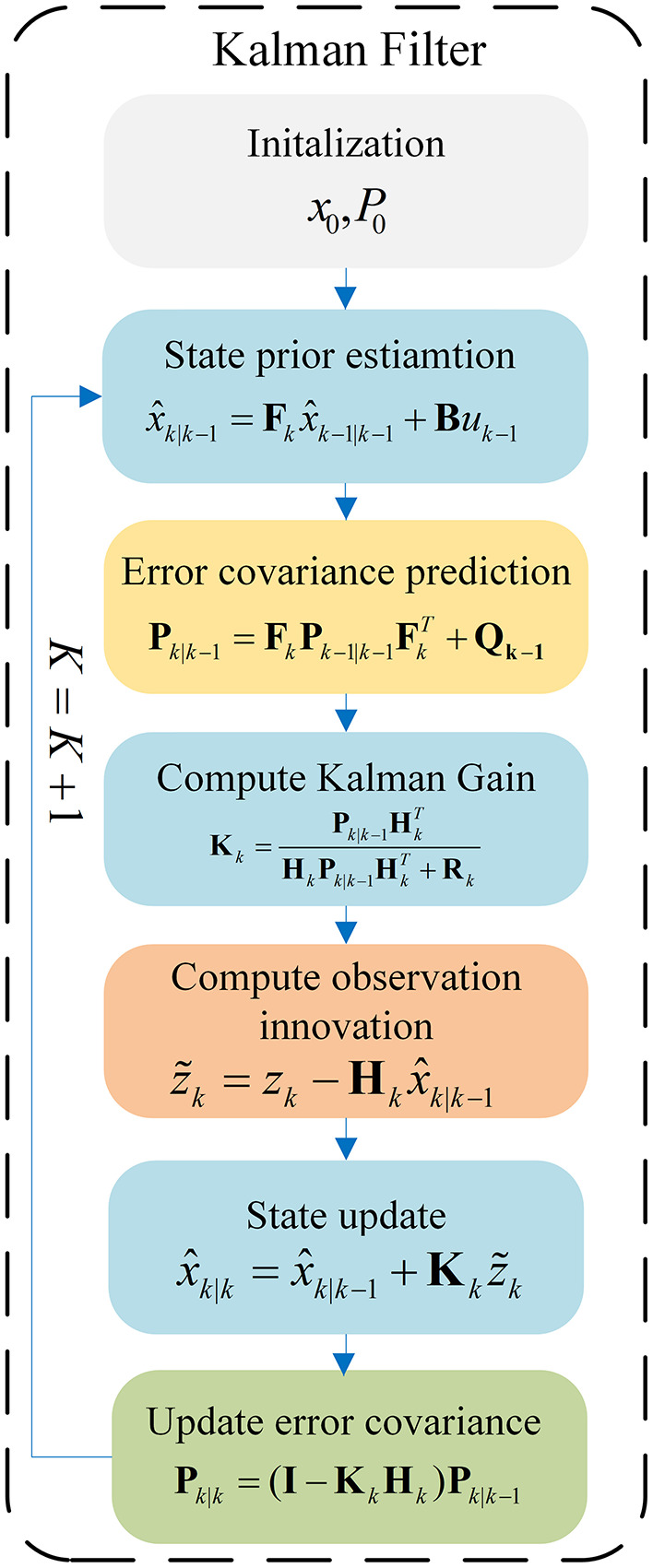
Information flow of the Kalman Filter.

In general, Recursive Kalman Filter can be divided into two steps: Prediction and Updation. The information flow of the Kalman Filter is shown in Figure 1. As shown in Figure 1, the predict step uses the state estimate from the previous timestep to produce an *priori* estimate of the state at the current timestep, which is expressed as follows:


(2) $$\begin{array}{l} {\hat{x}}_{k|k-1}={\text{F}}_{k}{\hat{x}}_{k-1|k-1}+\text{B}u_{k-1}\\ {\text{P}}_{k|k-1}={\text{F}}_{k}{\text{P}}_{k-1|k-1}{\text{F}}_{k}^{T}+{\text{Q}}_{k-1} \end{array}$$

In the update phase, the innovation between the current a *priori* estimation and the current observation information, is multiplied by the optimal Kalman gain and combined with the previous state estimate to optimal the state estimate. This improved estimate based on the current observation is termed a *posteriori* state estimate, which is summarized as follows:


(3) $$\begin{array}{l} {\text{K}}_{k}=\frac{{\text{P}}_{k|k-1}{\text{H}}_{k}^{T}}{{\text{H}}_{k}{\text{P}}_{k|k-1}{\text{H}}_{k}^{T}+{\text{R}}_{k}}\\ {\hat{x}}_{k|k}={\hat{x}}_{k|k-1}+{\text{K}}_{k}(z_{k}-{\text{H}}_{k}{\hat{x}}_{k|k-1})\\ {\text{P}}_{k|k}=(\text{I}-{\text{K}}_{k}{\text{H}}_{k}){\text{P}}_{k|k-1} \end{array}$$

Noise has a significant impact on the performance of a Kalman Filter system, as it directly affects the accuracy of the estimation. A Kalman Filter is designed to optimally combine observations and predictions in the presence of noise, which controls how the Kalman filter weights the model predictions versus the actual observations.

The process noise covariance **Q** represents the uncertainty in the model of the system dynamics. Higher values of **Q** means we have less reliability on the prediction and place more trust in the observations.

The observation noise covariance **R** means the uncertainty in the observations. Higher values of **R** suggest more noise in the observation, so the Kalman Filter will pay more attention to its predictions.

The following tuning steps are necessary before using the Kalman Filters:

- Set initial state vector ${\hat{x}}_{0}$.
- Set initial noise values for **Q** and **R**.
- Tuning the Process Noise Covariance **Q**.
- Tuning the Observation Noise Covariance **R**.
- Test the performance and adjust **Q** and **R**.

Although the noise parameters are tuned before using the Kalman Filters. It is difficult to obtain an accurate state transition model and observation model, especially in the nonlinear occasion, which results in a significant degradation of Kalman Filter performance.

### 2.2 Extended Kalman Filters

Differentiable nonlinear functions may be used in place of the state transition and observation models in the extended Kalman Filter:


(4) $$\{\begin{array}{l} {\hat{x}}_{k|k-1}=f({\hat{x}}_{k-1|k-1},u_{k-1})+w_{k-1}\\ z_{k}=h(x_{k})+v_{k}\\ w_{k-1}\sim N(0,{\text{Q}}_{k})\\ v_{k-1}\sim N(0,{\text{R}}_{k}) \end{array}$$

Similar to the linear Kalman Filter, *x*_*k*_ is the state vector of the system, *w*_*k*−1_ and *v*_*k*_ are process noise and observation noises respectively. Process noise is assumed to be drawn from a zero multivariate normal distribution N with covariance **Q**_*k*_. Observation noise is assumed to be zero mean Gaussian white noise with covariance **R**_*k*_.

Function *f* is used to predict the state from the previous estimation and function *h* is applied to produce the predicted measurement form the predicted state. Different from the linear Kalman Filter, the Jacobian of *f* and *h* are used to compute the covariance matrix in extended Kalman Filters.

At timestamp *k*, the Jacobian is evaluated with the current predicted states, thus it can be used in Kalman equations. The prediction procedure of EKF is presented as follows:


(5) $$\begin{array}{l} {\hat{x}}_{k|k-1}=f({\hat{x}}_{k-1|k-1},u_{k-1})\\ {\text{P}}_{k|k-1}={\text{F}}_{k}{\text{P}}_{k-1|k-1}{\text{F}}_{k}^{T}+{\text{Q}}_{\text{k}-1} \end{array}$$

The update procedure of EKF is calculated as follows:


(6) $$\begin{array}{l} {\text{K}}_{k}=\frac{{\text{P}}_{k|k-1}{\text{H}}_{k}^{T}}{{\text{H}}_{k}{\text{P}}_{k|k-1}{\text{H}}_{k}^{T}+{\text{R}}_{k}}\\ {\hat{x}}_{k|k}={\hat{x}}_{k|k-1}+{\text{K}}_{k}(z_{k}-h({\hat{x}}_{k|k-1}))\\ {\text{P}}_{k|k}=(\text{I}-{\text{K}}_{k}{\text{H}}_{k}){\text{P}}_{k|k-1} \end{array}$$

where the state transition function and observation model are defined as the following Jacobians:


(7) $$\begin{array}{l} \;F_{k}=\frac{\partial f}{\partial x}|{\hat{x}}_{k-1|k-1}\\ H_{k}=\frac{\partial h}{\partial x}|{\hat{x}}_{k|k-1} \end{array}$$

### 2.3 Transformer

#### 2.3.1 Transformer architecture

**Figure 2:**
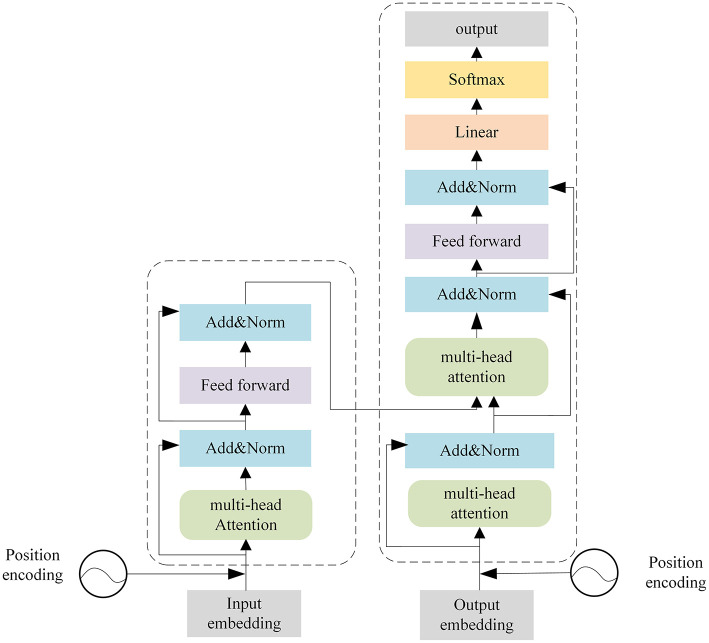
Architecture of the Transformer.

Transformer (Vaswani et al., 2017) was originally proposed in natural language processing and it has been applied in various sequence-to-sequence tasks. As shown in Figure 2, the Transformer is mainly composed of encoders and decoders with several basic transformer blocks. Transformer blocks inside the encoders and decoders remain in the same structure.

Encoders produce encodings for the input sequence, while the decoders take all the encodings from encoders and use contextual information to generate the prediction results. Each transformer block is composed of a multi-head attention layer, a feed-forward neural network, a skip connection connection, and a layer normalization operation.

#### 2.3.2 Self-attention mechanism

The Self-Attention Mechanism (SAM) is a core component of Transformer architecture, which seeks to emphasize the correlation between the input vector spaces.

As a first step, the input features are transformed into three different vectors using matrix multiplication, which is expressed as follows:


(8) $$\{\begin{array}{l} \text{Q}=F_{in}{\text{W}}_{Q}\\ \text{K}=F_{in}{\text{W}}_{K}\\ \text{V}=F_{in}{\text{W}}_{V} \end{array}$$

where **Q**, **K**, **V** are Query matrix, Key matrix, and Value matrix respectively. **W**_*Q*_, **W**_*K*_, **W**_*V*_ are used to generate the above-mentioned matrices. After that, attention map between different input vectors is calculated as follows:

- Compute scores between different input vectors with: **QK**^T^.
- Normalize the scores to improve the stability with: $\frac{\text{Q}{\text{K}}^{\text{T}}}{\sqrt{d_{k}}}$.
- Transform the scores into probabilities with softmax function: $\text{softmax}\left(\frac{\text{Q}{\text{K}}^{T}}{\sqrt{d_{k}}}\right)$.
- Generate the weighted value matrix with: $\text{softmax}\left(\frac{\text{Q}{\text{K}}^{T}}{\sqrt{d_{k}}}\right)\cdot V$.

**Figure 3:**
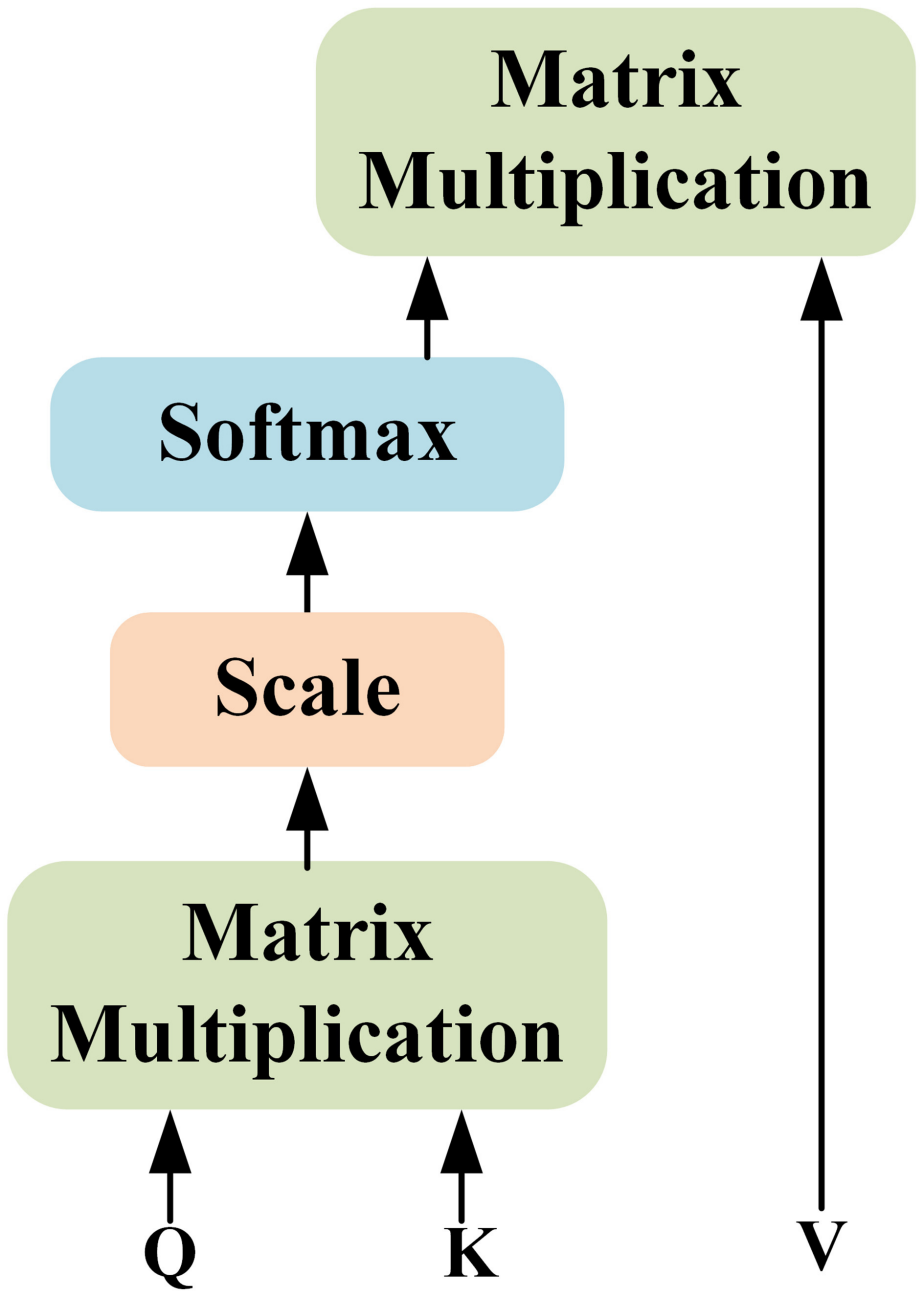
Illustration of the self-attention mechanism.

The above process can be describe with a single function:


(9) $$\text{Attention}(\text{Q},\text{K},\text{V})=\text{softmax}(\frac{\text{Q}{\text{K}}^{\text{T}}}{\sqrt{d_{k}}})\text{V}$$

where *d*_*k*_ means the dimension of the input. This procedure is shown in Figure 3.

#### 2.3.3 Position encoding

Transformer architecture can't guarantee the order of objects inside the sequence. Therefore, positional encoding is employed to assign a unique representation to each position inside the sequence. Cosine and sine functions are used to produce position encoding for varying frequencies, which is calculated as follows:


(10) $$\begin{array}{l} P\left(k,2i\right)=\sin\left(\frac{k}{n^{2i/d}}\right)\\ P\left(k,2i+1\right)=\cos\left(\frac{k}{n^{2i/d}}\right) \end{array}$$

where *K* is the position of an object inside the sequence, *d* means dimensions of the output embedding space, *P*(*k, j*) is position function, *n* is a predefine scalar, *i* is used to map column indices.

Using the position encoding, even positions correspond to a sine function and odd positions correspond to cosine functions.

## 3 Methodology

In this section, we present our KalmanFormer: a hybrid model and data-driven Kalman Filter for estimating the state of dynamic systems. Our KalmanFormer combines the model-based Kalman Filters with Transformer (Vaswani et al., 2017) to tackle model mismatch and non-linearities. As a first step, the information flow of our KalmanFormer will be presented. Subsequently, details information about the inputs for our KalmanFormer will be discussed. Following that, the architecture of the KalmanFormer and the training strategy will be introduced at the end of this section.

### 3.1 Information flow of KalmanFormer

**Figure 4:**
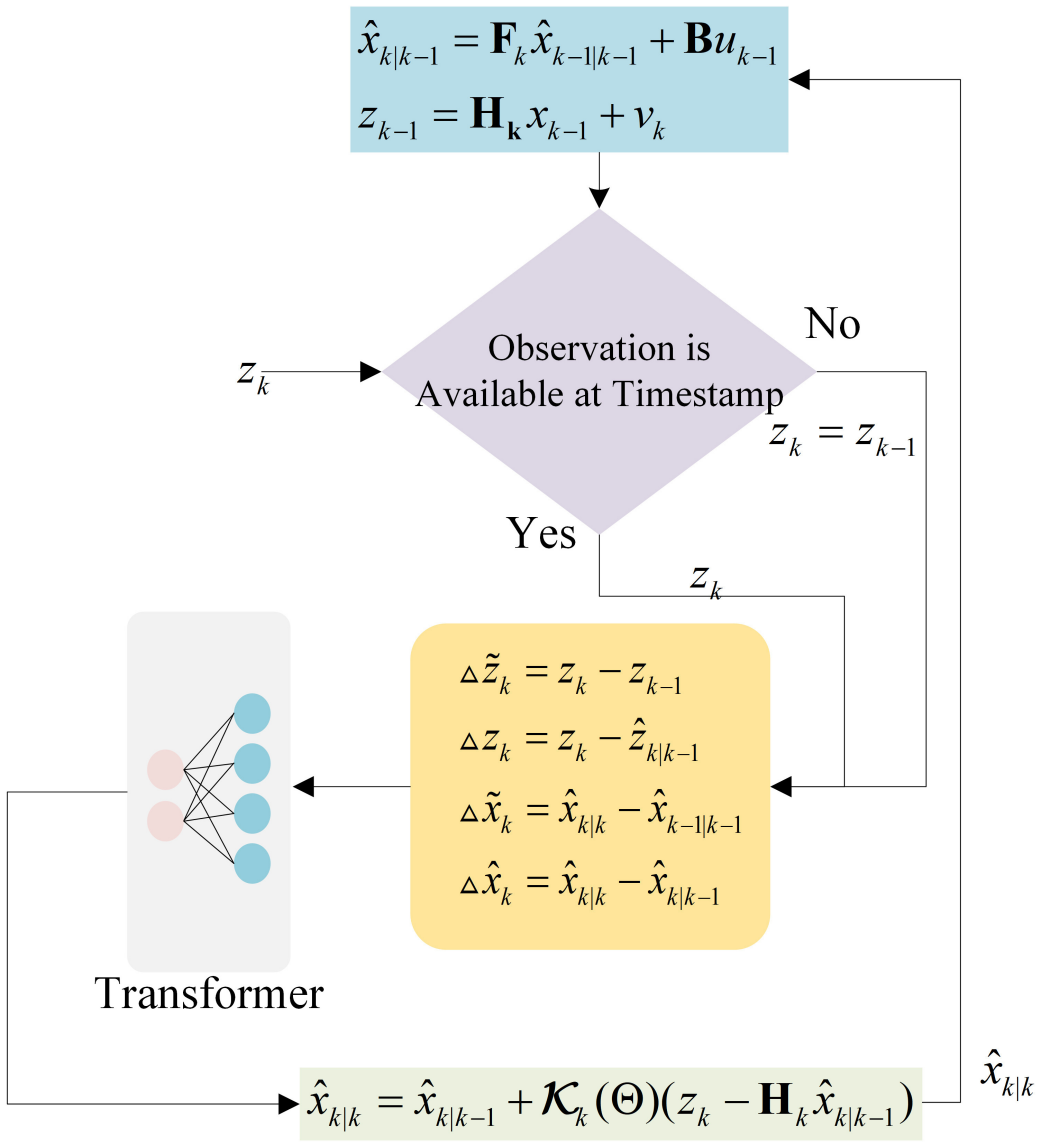
Information flow of the KalmanFormer.

In order to formulate our KalmanFormer, we identify the specific computation process of linear Kalman Filters that are based on unavailable knowledge. To be specific, the state transition model *F*_*k*_ and observation model *H*_*k*_ are available (although inaccurate), while the process noise **Q**_*k*_ and observation noise **R**_*k*_ are unavailable. As shown in Figure 1, unknown process noise and observation noise are used in Kalman Filters only for the purpose of calculating the Kalman Gain. To this end, we develop the KalmanFormer that tracks the Kalman Gain from the data and combines the learned Kalman Gain into the data flow of the Kalman Filter. The architecture of our KalmanFormer is provided in Figure 4. In the same manner as the model-based Kalman Filters, our KalmanFormer outputs the state estimate through two procedures: Prediction and Update.

1. In the prediction procedure, a *prior* state estimate of the current moment ${{\hat{x}}^{-}}_{k|k-1}$ is obtained from the previous *posterior* estimate ${\hat{x}}_{k-1|k-1}$.
2. In the update procedure, KalmanFormer uses the new observation *z*_*k*_ to compute the current state *posterior* ${\hat{x}}_{k|k}$ from the previous prior estimation ${\hat{x}}_{k|k-1}$, which is calculated in Equation 11. Instead of using the Kalman Gain matrix for the observation-update in the traditional Kalman Filters, KalmanFormer produces the Kalman Gain in a learned manner, denoted by $K_{K}\left(\text{Θ}\right)$, with the trainable parameters Θ:

(11) $$\begin{array}{l} {\hat{x}}_{k|k}={\hat{x}}_{k|k-1}+K_{K}\left(\text{Θ}\right)\left(z_{k}-H_{k}{\hat{x}}_{k|k-1}\right) \end{array}$$

### 3.2 Input features

The model-based Kalman Filters, including EKF, UKF, and CKF compute the Kalman Gain from the known statistical information. We use a Transformer to model the Kalman Gain in a learned fashion in this paper. To calculate the Kalman Gain, we have to provide the information to a deep neural network to use the information to calculate the Kalman Gain. Inspired by KalmanNet (Revach et al., 2022), we devise the following quantities, which can be used for the input of the KalmanFormer:

- *The observation difference*:${\tilde{z}}_{k}=z_{k}-z_{k-1}$
- *The innovation difference*: *z*_*k*_ = *z*_*k*_−ẑ_*k*|*k*−1_
- *The state evolution difference*: ${\tilde{x}}_{k}={\hat{x}}_{k|k}-{\hat{x}}_{k-1|k-1}$, which represents the difference between two consecutive posterior state estimate.
- *The state update difference*: ${\hat{x}}_{k}={\hat{x}}_{k|k}-{\hat{x}}_{k|k-1}$, which indicates the difference between the posterior state estimate and the prior state estimate.

Featurs ${\tilde{x}}_{k}$ and *z*_*k*_ indicate the uncertainty of the state estimates, while features *z*_*k*_ and ${\hat{x}}_{k}$ characterize the state transition and observation update process. Features *z*_*k*_ and ${\tilde{z}}_{k}$ contains the observation information, while features *x*_*k*_ and ${\hat{x}}_{k}$ characterize the states information of the system.

### 3.3 Details of the KalmanFormer

**Figure 5:**
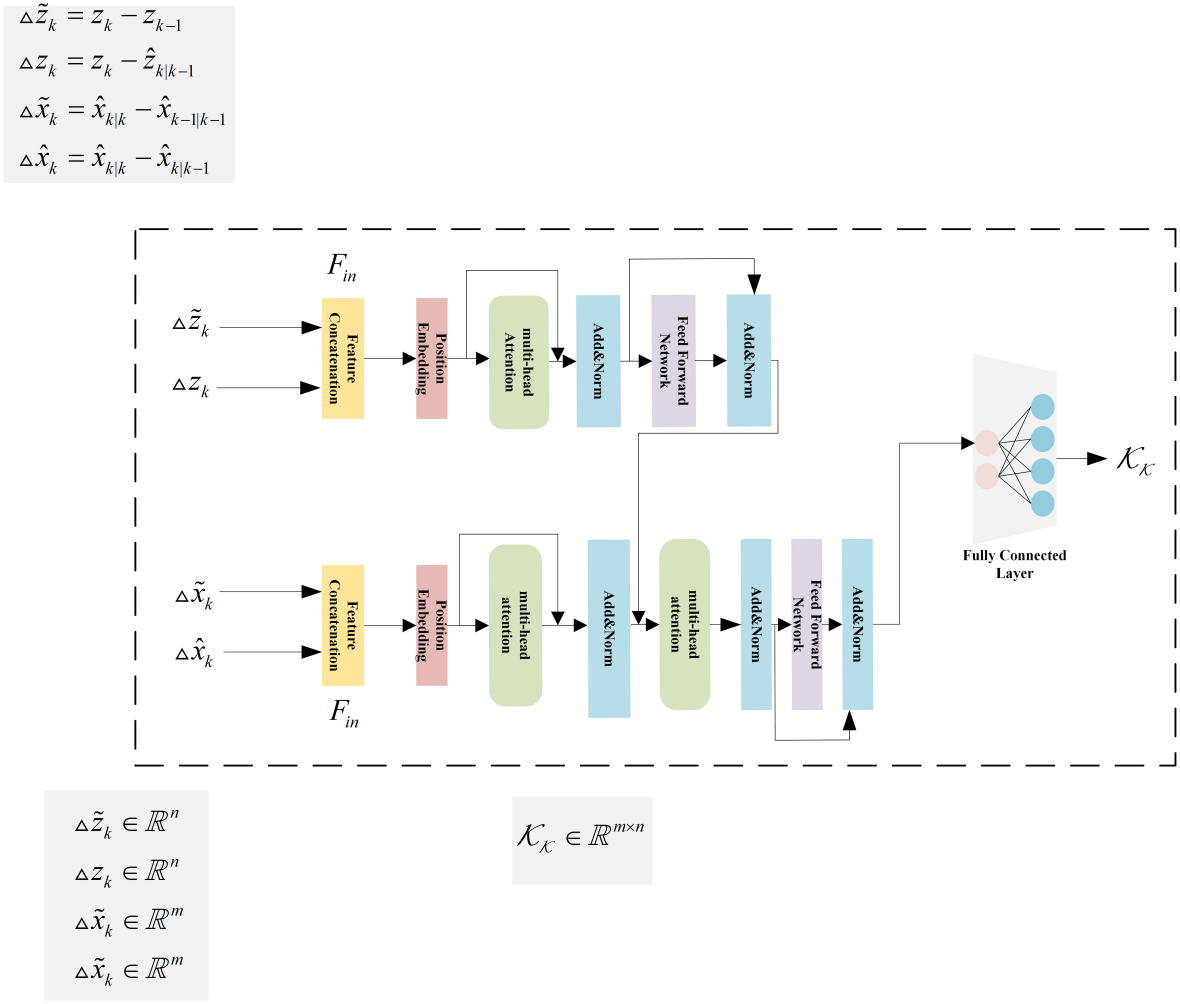
The details of the KalmanFormer.

The internal of KalmanFormer uses the features discussed in the previous section to compute the Kalman Gain. As a first step, we will introduce the input features of the Transformer. To be specific, ${\tilde{z}}_{k}$, *z*_*k*_, ${\tilde{x}}_{k}$, and ${\hat{x}}_{k}$ are used to construct our KalmanFormer. The data flow of the input features inside our KalmanFormer is shown in Figure 5.

Subsequently, the related observation features $\text{Δ}{\tilde{z}}_{k}\in {\mathbb{R}}^{n}$ and *z*_*k*_ ∈ ℝ^*n*^ are concatenate together to the input $F_{in}\in {\mathbb{R}}^{2n}$ of the Transformer encoders. And also, the related state features $\text{Δ}{\tilde{x}}_{k}\in {\mathbb{R}}^{m}$ and $\text{Δ}{\hat{x}}_{k}\in {\mathbb{R}}^{m}$ are concatenated together to the input for the Transformer decoders.

We devise three initial matrices $W_{\text{Q}}\in {\mathbb{R}}^{2m\times 2m}$, ${\text{W}}_{\text{K}}\in {\mathbb{R}}^{2m\times 2m}$, and ${\text{W}}_{V}\in {\mathbb{R}}^{2m\times 2m}$ to generate the **Q**, **K**, and **V** matrices, which is used to produce self-attention score described in Section 2.3.2.

Following is the Add and Norm operation. To be specific, Layer normalization is used to perform Add and Norm operation, which is expressed as follows:


(12) $$\begin{array}{l} L\text{ay}erNorm\left(X+attention\right) \end{array}$$

Then the feed forward neural network is used to generate output, which is presented as:


(13) $$FFN=\text{ReLU}({\text{XW}}_{1}+b_{1}){\text{W}}_{2}+b_{2}$$

The feed-forward neural network is composed of two layers of the fully connected network. The W_1_ and W_2_ are the weights for the two layers of network. *b*_1_ and *b*_2_ are the bias. ReLU is the Rectified Linear Unit activate function.

Then the output of the Transformer encoder and the concatenated state input features to produce the learned Kalman Gain.

In our implementation, the input dimension is set to 4, the feed-forward dimension is set to 64, and 2 heads are employed in the Multi-head Self Attention Mechanism (MHSA). Furthermore, we stack the encoder and decoder 2 times to produce the learned Kalman Gain.

The information flow of the KalmanFormer is illustrated in Figure 5.

### 3.4 Training algorithm

A supervised learning paradigm is used to train the KalmanFormer using the available labeled data. Instead of producing the *posterior* estimate state, our KalmanFormer produces the Kalman Gain. Consequently, we define (Equation 21) to backpropagate the loss of Kalman Gain to train our KalmanFormer:


(14) $$\begin{array}{l} \frac{\partial L}{\partial K_{k}}=\frac{\partial ||K_{k}\text{Δ}z_{k}-\text{Δ}x_{k}{||}^{2}}{\partial K_{k}}=2\cdot \left(K_{k}\text{Δ}z_{k}-\text{Δ}x_{k}\right)\cdot \text{Δ}z_{k}^{T} \end{array}$$

where $\text{Δ}x_{k}=x_{k}-{\hat{x}}_{k|k-1}$. The Equation 14 indicates that we can learn the computation of the Kalman Gain by training KalmanFormer end-to-end using the squared-error loss.

In general, the dataset used for training the KalmanFormer consists of *N* length *T*trajectories. Let *T* denote the length of *i*-th training trajectory inside the dataset. The dataset can be expressed by $D={\left\{\left({\text{Z}}_{i},{\text{X}}_{i}\right)\right\}}_{1}^{N}$, where


(15) $$\begin{array}{l} Z_{i}=\left[z_{1}^{\left(i\right)},z_{2}^{\left(i\right)}...,z_{T}^{\left(i\right)}\right],X_{i}=\left[x_{0}^{\left(i\right)},x_{1}^{\left(i\right)}...,x_{T}^{\left(i\right)}\right] \end{array}$$

The empirical loss function for the *i*-th trajectory training inside the dataset is defined as follows:


(16) $$\begin{array}{l} l_{i}\left(\text{Θ}\right)=\frac{1}{T_{i}}\overset{T_{i}}{\underset{k=1}{\sum }}||{\text{Ψ}}_{\text{Θ}}\left({\hat{x}}_{k-1}^{i},z_{k}^{\left(i\right)}\right)-x_{k}^{\left(i\right)}{||}^{2}+\xi \cdot ||\text{Θ}{||}^{2} \end{array}$$

where Ψ_Θ_ represents the output of our KalmanFormer, Θ is the trainable parameters inside the KalmanFormer, and ξ is regularization coefficient. Let $\text{Δ}{\text{x}}_{k}^{\left(k\right)}={\text{x}}_{k}^{\left(k\right)}-{\hat{\text{x}}}_{k|k-1}^{\left(k\right)}$ and $\text{Δ}z_{k}^{\left(k\right)}=z_{k}^{\left(k\right)}-{ẑ}_{k|k-1}^{\left(k\right)}$ be the state prediction error and the measurement innovation at timestamp *k*. The partial derivative of the loss function respective to the Kalman gain matrix is:


(17) $$\begin{array}{l} \frac{\partial l\left(\text{Θ}\right)}{\partial K_{k}\left(\text{Θ}\right)}=\frac{1}{LT_{k}}\overset{L}{\underset{k=1}{\sum }}\overset{T_{k}}{\underset{k=1}{\sum }}\frac{\partial ||\text{Δ}x_{k}^{\left(k\right)}-K_{k}\left(\text{Θ}\right)\text{Δ}z_{k}^{\left(k\right)}{||}_{2}^{2}}{\partial K_{k}\left(\text{Θ}\right)} \end{array}$$

By plugging into the chain rule:


(18) $$\begin{array}{l} \frac{\partial l\left(\text{Θ}\right)}{\partial \left(\text{Θ}\right)}=\frac{\partial l\left(\text{Θ}\right)}{\partial K_{k}\left(\text{Θ}\right)}\frac{\partial K_{k}\left(\text{Θ}\right)}{\partial \left(\text{Θ}\right)} \end{array}$$

We can adopt a stochastic gradient descent algorithm to optimize Θ by using $\frac{\partial l\left({\text{Θ}}^{*}\right)}{\partial \left({\text{Θ}}^{*}\right)}=0$.

## 4 Numerical experiments

In this section, we design a series of experiments to evaluate the performance of our proposed KalmanFormer and compare it to some other benchmarks. As a first step, we make a brief description of the training setup of our KalmanFormer. Following that, we conduct the simulation experiments including nonlinear cases to evaluate the performance of our proposed method. At the end of this section, IMU and GPS information are employed to investigate the effectiveness of our proposed method.

### 4.1 Implement details

To be specific, the dimensions of concatenated observation difference and innovation difference are 8, which is the input to the encoder for the transformer. Also, the dimensions of state evolution difference and state update difference are 4, which is the input to the decoders of Transformer. The feed-forward dimension inside the encoder and decoder is 64, and 2 heads are employed in the multi-head attention mechanism. Furthermore, we stack the encoder and decoder 2 times to produce the output. After the output is obtained, a fully connected layer is used to generate the learned Kalman Gain.

Furthermore, we conduct all of our training and validation experiments on the Pytorch (Paszke et al., 2019) platform using a single RTX 3090 GPU card, CUDA11.6, and cuDNN version 8. Furthermore, the Cosine Annealing Schedule is employed to adjust the learning rate in the training procedure, which can be expressed as follows:


(19) $$\begin{array}{l} \eta_{t}=\eta_{\min}+\frac{1}{2}\left(\eta_{max}-\eta_{min}\right)\left(1+\cos\left(\frac{T_{cur}}{T_{max}}\pi \right)\right) \end{array}$$

where η_*t*_ represents the learning rate of the current iteration, η_min_ and η_max_ mean the predefined minimum and maximum learning rate respectively. *T*_*cur*_ and *T*_*max*_ are the current iteration and maximum iterations respectively.

Adam (Kingma and Ba, 2014) optimizer is used to train the KalmanFormer. Different hyper parameters are performed on the simulation and multi-sensor fusion experiments. The specific information about the hyperparameters is shown in Table 1.

**Table 1:** Details information about the hyperparameters.

**Experiment type**	**Epochs**	**Batch size**	**Learning rate**	**Weight decay**
Simulation	200	30	1e-3	1e-3
Multi-sensor fusion	100	10	1e-3	1e-4

### 4.2 Simulation experiments

In this section, a series of simulation experiments are designed to demonstrate the effort of our proposed KalmanFormer. We make a comparison with EKF and KalmanNet (Revach et al., 2021).

#### 4.2.1 Test metric

Mean Square Error (MSE) is used to evaluate the effect of our proposed KalmanFormer, which is computed as follows:


(20) $$\begin{array}{l} MSE=\frac{1}{N}\overset{N}{\underset{j=1}{\sum }}\overset{T}{\underset{i=1}{\sum }}|{\left(x_{est}-x_{true}\right)}_{i}{|}^{2} \end{array}$$

where *x*_*est*_ means the output from our KalmanFormer, *x*_*true*_ represents corresponding ground-truth. *N* means the number of the testing trajectories. *T* is the length of current trajectory.

#### 4.2.2 Non-linear Lorenz attractors

The Lorenz attractor (Tucker, 1999) describes a non-linear chaotic system used for atmospheric convection. The Lorenz system is expressed by following three differential equations that define the convection rate, the horizontal temperature variation, and the vertical temperature variation of a fluid:


(21) $$\begin{array}{l} \frac{\partial z_{1}}{\partial t}=10\left(z_{2}-z_{1}\right),\frac{\partial z_{2}}{\partial t}=z_{1}\left(28-z_{3}\right)-z_{2},\frac{\partial z_{3}}{\partial t}=z_{1}z_{2}-\frac{8}{3}z_{3}, \end{array}$$

**Figure 6:**
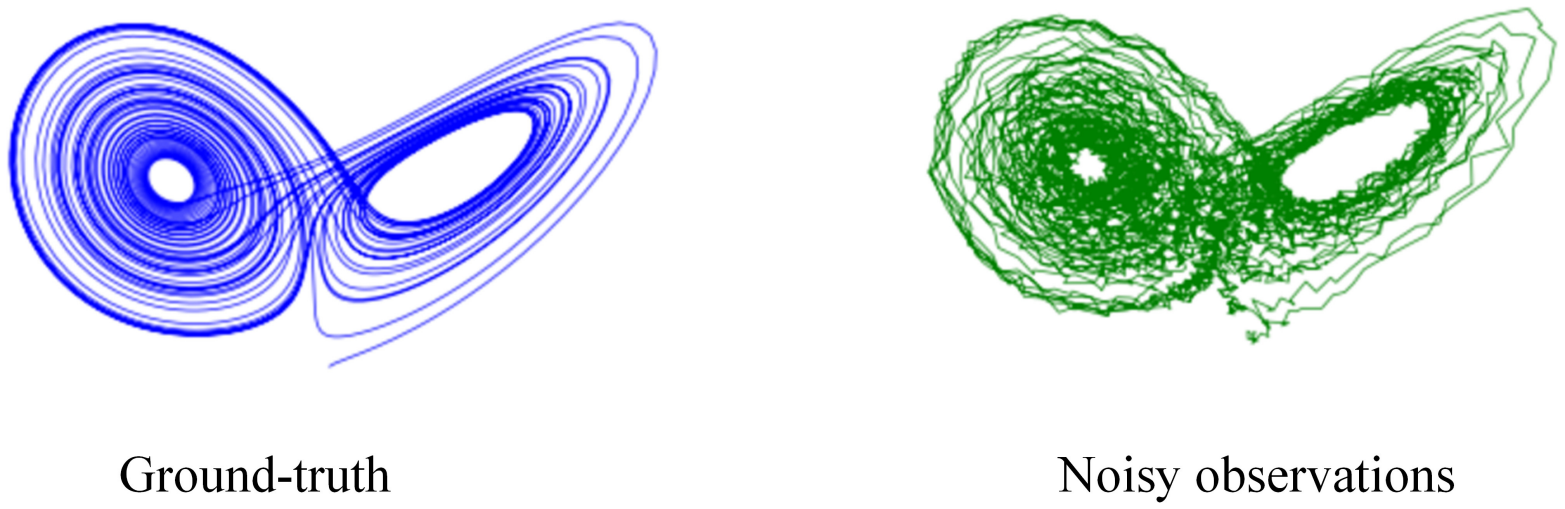
Illustration of trajectories.

In order to generate the simulated trajectories, we run the Lorenz equations described at Equation 21 with a time step of Δ*t* = 0.05 and add Gaussian noise of standard deviation σ = 0.05 to the results. The noisy data is considered as the measurements while the decimated data is regarded as the ground truth trajectory for our experiments. The trajectories of ground truth and noisy observations are shown in Figure 6.

Assuming a three-dimensional vector $x={\left[z_{1},z_{2},z_{3}\right]}^{\text{T}}\in \text{R}$, the dynamic matrix *A*(*x*) of the system from Equation 21 is expressed as follows:


(22) $$\begin{array}{l} A\left(x\right)=\left[\begin{array}{ccc} -10 & 10 & 0\\ 28-z_{3} & -1 & 0\\ z_{2} & 0 & -\frac{8}{3} \end{array}\right]\left[\begin{array}{c} z_{1}\\ z_{2}\\ z_{3} \end{array}\right] \end{array}$$

After that, Taylor expansion is used to obtain the state transition function:


(23) $$\begin{array}{l} F_{k}\left(x_{k}\right)=I+\overset{J}{\underset{j=1}{\sum }}\frac{{\left(A\left(x_{k}\right)k\right)}^{j}}{j!} \end{array}$$

where *I* represents the identity matrix and *J* means the number of Taylor expansion. We set J = 5 in our experiments. For the measurement model, we set *H* = *I*. For the noise parameters, we set **Q** = q^2^**I, R** = *r*^2^**I**, where q=0.8, r=1.

### 4.3 Multi-sensor information fusion

We further evaluate the effectiveness of the proposed KalmanFormer in multi-sensor fusion. We employ the Michigan NCLT dataset (Carlevaris-Bianco et al., 2016) with different types of sensors to perform our experiments.

The NCLT dataset was obtained from a mobile robot platform equipped with various sensors, including Real Time Kinematic GPS, IMU, Consumer-grade GPS, etc. In our experiments, IMU is employed to provide angular speed information and acceleration information, which is used to design the state transition function. The consumer-grade GPS is applied to provide the observation of the displacement. The Real-Time Kinematic GPS is used to generate a more accurate state of the system, which is used to evaluate the effectiveness of the proposed method.

#### 4.3.1 Coordinates definition

A North-East-Down (NED) frame is employed to describe the robot's pose and position. Furthermore, the fixed origin point of the NED frame is shown in Table 2.

**Table 2:** Origin point information for NED frame.

**Latitude origin**	**42.29322deg**
Longitude origin	–83.709657 deg
Altitude origin	270 m

The angular and acceleration information from the IMU is measured in the IMU's reference frame, which closely aligns with the robot's reference coordinate. It is necessary to transform the IMU reading from IMU's frame into a global frame.

**Figure 7:**
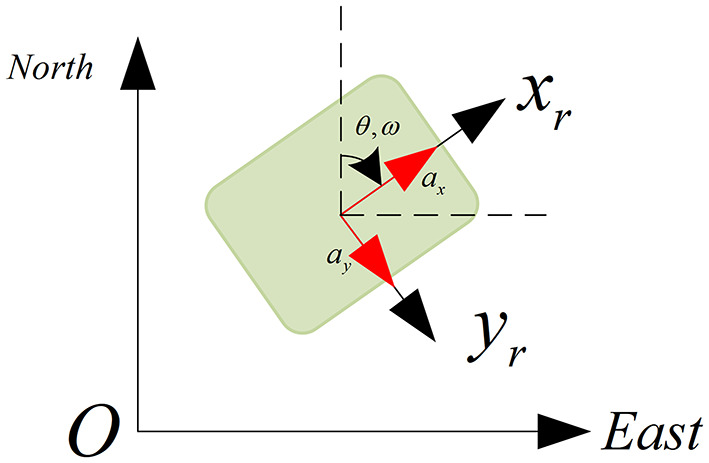
Transformation between IMU frame and Global Frame (NED).

As shown in Figure 7, we can obtain the transformation between the IMU frame and the global frame, which is calculated as:


(24) $$\{\begin{array}{l} a_{gx}=a_{x}\cos(-\theta )-a_{y}\sin(-\theta )\\ a_{gy}=a_{x}\sin(-\theta )-a_{y}+\cos(-\theta ) \end{array}$$

#### 4.3.2 State transition model

The state vector of the system in the global coordinate is defined as:


(25) $$\begin{array}{l} x_{k}\text{=}\left[x,y,v_{x},v_{y},\theta ,\omega \right] \end{array}$$

where *x*_*k*_, *y*_*k*_ represent the position of the robot in the global frame. *v*_*k*_ and *v*_*y*_ represent the velocities. θ and ω mean the heading angle and angular velocities respectively.

In the global coordinate system, the state transition model takes the IMU's readings, including heading θ, angular velocity ω, and the accelerations as the control input. The state transition model (in the NED frame) is then:


(26) $$\begin{array}{l} {\hat{x}}_{k|k-1}=F_{k}\left(x_{k-1},u_{k-1}\right)=\left[\begin{array}{c} \begin{array}{c} x_{k-1}+v_{x}\text{Δ}k+\frac{1}{2}a_{gx}\text{Δ}k^{2}\\ y_{k-1}+v_{y}\text{Δ}k+\frac{1}{2}a_{gy}\text{Δ}k^{2}\\ v_{x-1}+a_{gx}\text{Δ}k \end{array}\\ v_{y-1}+a_{gy}\text{Δ}k\\ \theta_{k}\\ \omega_{K} \end{array}\right] \end{array}$$

#### 4.3.3 Observation model

The GPS observation model produces a prediction of the expected GPS observation based on the predicted state. Here we use the consumer-grade GPS to produce the observation of the displacement. The observation model is expressed as follows:


(27) $$\begin{array}{l} z_{k|k-1}=H_{k}{\hat{x}}_{k|k-1}=\left[\begin{array}{cccccc} 1 & 0 & 0 & 0 & 0 & 0\\ 0 & 1 & 0 & 0 & 0 & 0 \end{array}\right]{\hat{x}}_{k|k-1} \end{array}$$

#### 4.3.4 Noise setting

The initial process noise **Q**_*k*_ and measurement noise **R**_*k*_ matrices of the EKF are expressed in Equations 2, 3. These **Q**_*k*_ and **R**_*k*_ matrices are determined using empirical data as well as completing experimental tuning. The initial **Q**_*k*_ and **R**_*k*_ matrices used in our experiments are developed as follows:


(28) $$\begin{array}{l} Q_{k}=\left[\begin{array}{cccccc} 1 & 0 & 0 & 0 & 0 & 0\\ 0 & 1 & 0 & 0 & 0 & 0\\ 0 & 0 & 10000 & 0 & 0 & 0\\ 0 & 0 & 0 & 10000 & 0 & 0\\ 0 & 0 & 0 & 0 & 0.001 & 0\\ 0 & 0 & 0 & 0 & 0 & 0.01 \end{array}\right] \end{array}$$

(29) $$\begin{array}{l} R_{k}=\left[\begin{array}{cc} 100 & 0\\ 0 & 100 \end{array}\right] \end{array}$$

### 4.4 Model mismatch

#### 4.4.1 State transition model mismatch

We devise experiments to investigate the robustness of the KalmanFormer when the state transition model is mismatched. This is achieved by using three 3-dimensional rotation matrices:


(30) $$\begin{array}{l} RZ=\left[\begin{array}{ccc} \cos\left(yaw\right) & -\sin\left(yaw\right) & 0\\ \sin\left(yaw\right) & \cos\left(yaw\right) & 0\\ 0 & 0 & 1 \end{array}\right] \end{array}$$

(31) $$\begin{array}{l} RY=\left[\begin{array}{ccc} \cos\left(pitch\right) & 0 & \sin\left(pitch\right)\\ 0 & 1 & 0\\ -\sin\left(pitch\right) & 0 & \cos\left(pitch\right) \end{array}\right] \end{array}$$

(32) $$\begin{array}{l} RX=\left[\begin{array}{ccc} 1 & 0 & 0\\ 0 & \cos\left(roll\right) & -\sin\left(roll\right)\\ 0 & \sin\left(roll\right) & \cos\left(roll\right) \end{array}\right] \end{array}$$

(33) $$\begin{array}{l} yaw=roll=pitch=1^{{}^{\circ}},5^{{}^{\circ}} \end{array}$$

We evaluate the performance in the condition of model mismatch real-word NCLT datasets. The mismatched state transition real-world is expressed as follows:


(34) $$\begin{array}{l} F_{real}^{\text{rotated}}=RX•RY•RZ•\left[\begin{array}{ccc} 1 & \text{Δ}k & \frac{1}{2}\text{Δ}k^{2}\\ 0 & 1 & \text{Δ}k\\ 0 & 0 & 1 \end{array}\right] \end{array}$$

where $F_{real}^{\text{rotated}}$ is the mismatched state transition model for the real-world experiments. In our experiments, the rotation angle is set to 1° and 5°to verify the model performance.

#### 4.4.2 Observation model mismatch

Additionally, we investigate the performance of our proposed KalmanFormer with EKF when the observation function is mismatched. The mismatched observation function is expressed as follows:


(35) $$\begin{array}{l} H_{rotated}=H•RX•RY•RZ \end{array}$$

We set the rotation angle to 10° to validate the effectiveness on the simulation dataset.

Furthermore, we transform the observation in Cartesian coordinates into Spherical coordinates using the equation and compare the performance.


(36) $$\{\begin{array}{c} r=\sqrt{z_{1}^{2}+z_{2}^{2}+z_{3}^{2}}\\ \theta =\cos^{-1}(\frac{z_{3}}{r})\\ \phi =\tan^{-1}(\frac{z_{2}}{z_{1}}) \end{array}$$

### 4.5 Evaluation results

In this section, we will discuss the performance of our proposed KalmanFormer with EKF and KalmanNet for both linear and non-linear systems. Furthermore, we will investigate the performance of the proposed KalmanFormer using the NCLT dataset.

#### 4.5.1 Simulation results

**Figure 8:**
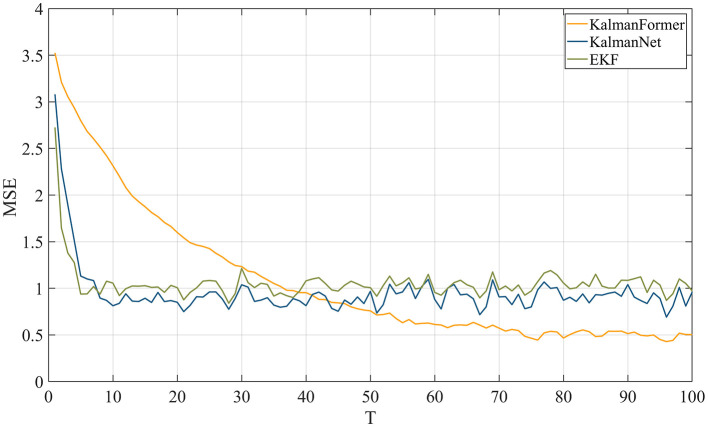
MSE comparison results with KalmanNet and EKF on synthetic dataset.

MSE metric is used to demonstrate the effectiveness of the proposed KalmanFormer in non-linear Lorenz attractors. As shown in Figure 8, our KalmanFormer achieves a higher MSE result in the first 30 points of the Test sequence when compared to KalmanNet and EKF. However, after the 40 points, the MSE of our KalmanFormer is much lower than EKF and KalmanNet.

Besides that, Euclidean Distance is used to evaluate the effectiveness of our methodology over the whole test trajectories. Euclidean Distance is expressed as follows:


(37) $$\begin{array}{l} \text{distance}=\overset{N}{\underset{i=1}{\sum }}\sqrt{\overset{T}{\underset{j=1}{\sum }}{\left(x_{est}^{j}-x_{true}^{j}\right)}^{2}} \end{array}$$

where $x_{true}\in [\begin{array}{ccc} z_{1} & z_{2} & z_{3}{]}^{T} \end{array}\in \mathbb{R}$ means the ground truth of the state vector. $x_{\text{est}}\in [\begin{array}{ccc} z_{1} & z_{2} & z_{3}{]}^{T} \end{array}\in \mathbb{R}$ represents the estimation from our KalmanFormer. *N* is the number of the trajectories. *T* is the length for each trajectory.

Using Equation 37, the distance of our proposed method is 136. While the distance of KalmanNet is 209, which demonstrates the superiority of our KalmanFormer. In conclusion, KalmanFormer achieves more accurate performance on the Simulation Test set.

**Figure 9:**
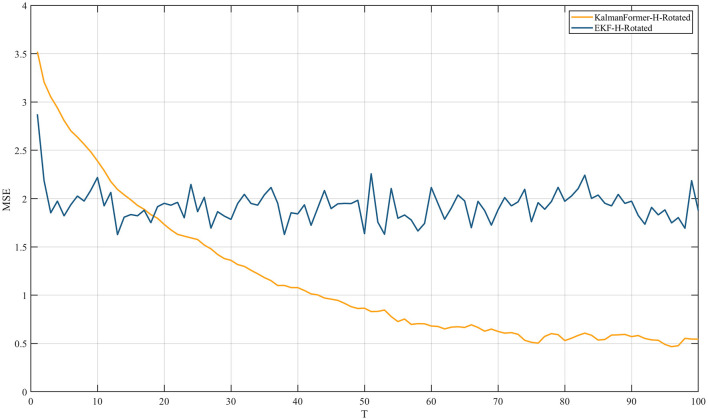
MSE comparison results with EKF when the observation is mismatched.

Additionally, Figure 9 reports the experiment results when the observation model is mismatched.

We can observe that the proposed KalmanFormer achieves lower MSE performance than EKF in the same experiment setup when the observation model is disturbed by the rotation matrix.

**Figure 10:**
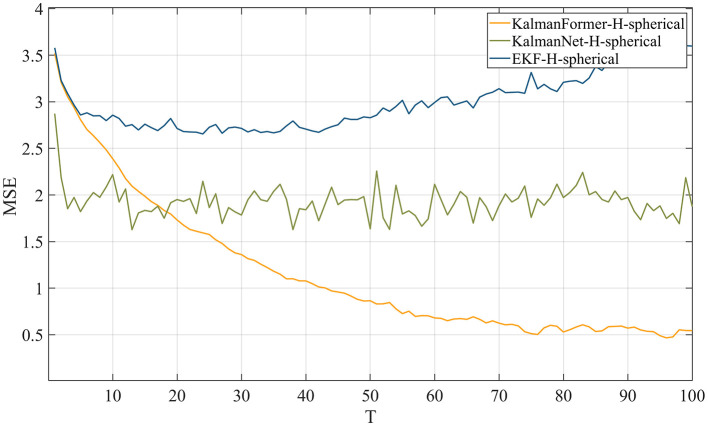
MSE comparison results with EKF when the observation is in spherical coordinate.

Figure 10 reports the results when the observation in transformed into spherical coordinates. We can see that our proposed KalmanFormer achieves the best performance compared to KalmanNet and EKF in the condition of the mismatched observation.

Finally, we compare the time complexity of the KalmanFormer compared to EKF and KalmanNet through simulation experiments. Parameters, storage space, and inference time are adopted to verify the computational complexity of the KalmanFormer, KalmanNet, and EKF. The inference time is computed on the simulation experiments. The comparison results are shown in Table 3.

**Table 3:** The complexity comparison results on simulation experiments.

**Method**	**Parameters**	**Storage (KB)**	**Inference time (s)**
KalmanFormer	8,081	66	21
KalmanNet	23,928	46	19
EKF	\	\	20

As shown in Table 3, the KalmanNet and the KalmanFormer have similar space demand and the similar running speeds on the simulation experiments. To be specific, the KalmanNet needs 44 KB harddisk space to store while the KalmanFormer 66KB needs disk space. Furthermore, we compare the inference time on the whole dataset. The inference time of the EKF is about 20s while the KalmanFormer runs about 21s. We can conclude that the proposed KalmanFormer has a similar time complexity with the EKF and KalmanNet and it can be further used in real-world applications.

#### 4.5.2 Multi-sensor information fusion

**Figure 11:**
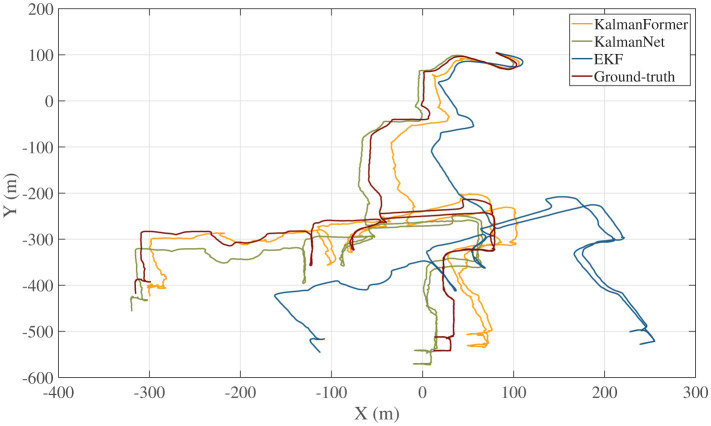
Trajectory comparison results with KalmanNet and EKF.

The trajectory we used for training and validating KalmanFormer and KalmanNet are obtained from the date of 2012-01-22 within the NCLT datasets. Furthermore, a date of 2012-04-29 trajectory is used to test the performance. The sample rate of the training, validation, and test is 1 *HZ*. The trajectory comparison result is shown in Figure 11.

**Figure 12:**
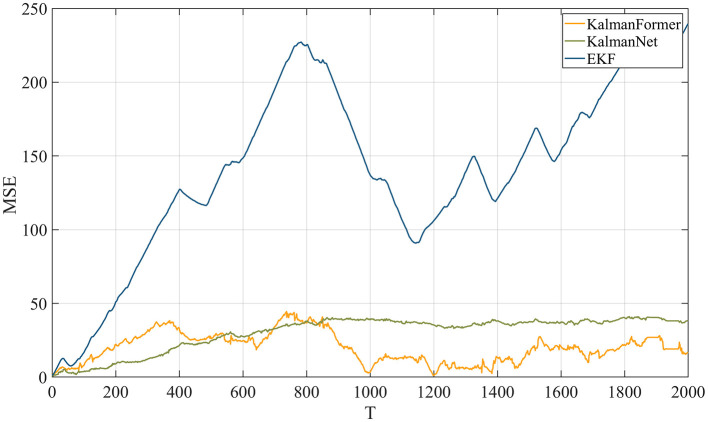
MSE performance with KalmanNet and EKF on NCLT dataset.

As shown in Figure 11, our KalmanFormer performs better than EKF and KalmanNet. In order to evaluate the property of our KalmanFormer, we make a comparison with EKF and KalmanNet in terms of MSE using the same data in Figure 11. The result is shown in Figure 12. According to Figure 12, our KalmanFormer achieves similar accuracy at the first 500 points of the testing set. However, in the last 1,500 points, our method achieves better performance in MSE compared to KalmanNet.

Additionally, Equation 37 is used to evaluate the validity over the whole test trajectory quantitatively. The distance of KalmanFormer is 19 m, the distance of KalmanNet is 30 m, and the distance of EKF is 316 m, which proves the superiority of the KalmanFormer.

**Figure 13:**
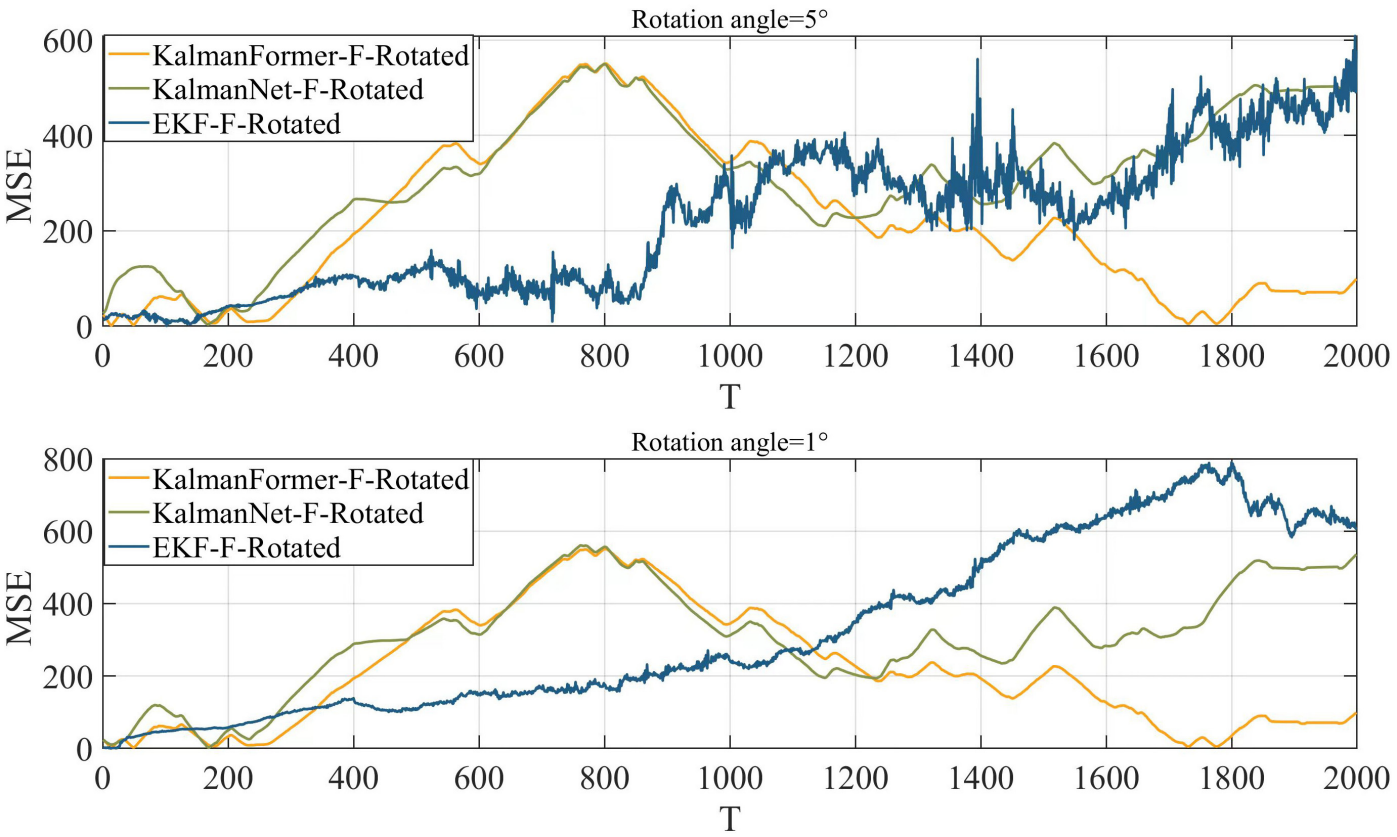
MSE performance when the state transition model is mismatched.

Finally, we investigate the results using the mismatched state transition function with the rotation angles of 1° and 5°. Figure 13 reports the results when the state transition function is mismatched. We can observe that when the state transition model is disturbed by the rotation matrix with a rotation angle of 1°, our KalmanFormer has a similar performance to the KalmanNet and outperforms the EKF. When the rotation angle is set to 5°, the performance of EKF degrades significantly. And the KalmanFormer outperforms the KalamNet and EKF. Even our KalmanFormer achieves lower MSE than KalmanNet.

## 5 Conclusion

In this paper, we proposed KalmanFormer, which is a hybrid of data-driven and model-driven implementation of the Kalman Filters. KalmanFormer incorporates a Transformer architecture within the learning process of computing the Kalman Gain (KG) and combines the learned KG into a traditional Kalman Filter. The proposed KalmanFormer uses the Kalman Filter without requiring any prior knowledge of process statistics or measurement noise statistics, even if the system model is mismatched. It has been demonstrated through numerical experiments that KalmanFormer is capable of achieving the minimum MSE when properly trained. It has also been proven that KalmanFormer is more robust to inaccurate knowledge of state space parameters in multi-sensor information fusion.

## Data Availability

The public NCLT dataset is used in this study. Researchers can get them from website of https://robots.engin.umich.edu/nclt/.
